# Protocol for exploring pathways to equitable outcomes in post-stroke aphasia and dysphagia

**DOI:** 10.1371/journal.pone.0308963

**Published:** 2024-09-27

**Authors:** Charles Ellis, Marcello Perraillon, Richard Lindrooth, Molly Jacobs, Karen Hegland, Anouk Grubaugh, Candice Adams-Mitchell

**Affiliations:** 1 Department of Speech Language & Hearing Sciences, University of Florida, Gainesville, FL, United States of America; 2 Department of Health Systems, Management & Policy, University of Colorado Anschutz Medical Campus, United States of America; 3 Department of Health Services, Research, Management and Policy, University of Florida, Gainesville, FL, United States of America; 4 Department of Psychiatry & Behavioral Sciences, Medical University of South Carolina, United States of America; Washington University in St Louis, UNITED STATES OF AMERICA

## Abstract

**Introduction:**

Longstanding racial disparities in stroke-related outcomes have been well documented. However, the underlying causes of observed disparities have neither been clearly determined nor have strategies to mitigate disparities been developed. Evidence suggests that racial disparities may be partially explained by structural barriers that can arise from implicit and explicit provider biases, institutional practices, public policies, or characteristics of the community where patients reside and recover from their conditions. The objective of this study is to move beyond traditional measures of disparities by identifying th**e** mechanisms that drive these observed disparities in aphasia and dysphagia across the continuum of care. In this study we will follow stroke survivors for 12 months post-discharge, which will allow us to examine the patient, provider, health system, and administrative factors that impact their aphasia and dysphagia recovery.

**Methods:**

This study will utilize a 100% sample of Medicare fee-for-service claims data for persons hospitalized for stroke. Patients discharged from acute stroke care will be followed for at least 12 months to measure the timing of post-acute care transition(s) and post-acute care speech-language pathology (SLP) utilization. Functional communication and swallowing outcomes will be measured at initiation, conclusion of post-acute care treatment, and points in-between allowing us to link improvement of functional communication (i.e., aphasia) and swallowing ability (i.e., dysphagia) to aphasia/dysphagia treatments as patients transition through post-acute settings. Then, using regression decomposition methods, we will examine the relationships between race and: (a) where patients receive treatment, (b) the timing of transition between sites of care, and (c) the quality of care received. Decomposition methods will allow us to elucidate the multiple factors that contribute to underlying observed health disparities by quantifying the extent to which differences between the outcomes of two groups are explained by 1) differential endowments or characteristics, such as geographic access, education, risk factors, or insurance coverage; or 2) differences in decision-making, defined as between group differences in outcomes despite equal endowments or unmeasured differences.

**Discussion:**

We hypothesize that racial disparities in aphasia and dysphagia outcomes will not only result from differences in the quantity and timing of services provided in the post-acute and community settings, but also structural differences at the community level. These findings will provide a more comprehensive understanding of healthcare use and outcomes.

## Introduction

Studies consistently show that Non-Hispanic Blacks (Blacks) experience stroke at a rate of 2–3 times that of Non-Hispanic Whites (Whites) [1]. While these differences have been well-documented, there is still limited evidence on the causes of observed disparities and how they can be mitigated. Recent research suggests that disparities in health-related outcomes can be partially explained by structural barriers that arise from implicit and explicit provider biases, institutional practices, public policies, and the community characteristics where patients reside and recover from their conditions [2]. Despite these findings, less attention has been given to the complexity of factors that emerge from individuals, institutions, and/or communities and how they impact the availability, accessibility, and quality of post-stroke rehabilitation care. Interestingly, access to care for stroke and related chronic conditions in the United States (US) has dramatically improved over time, yet there is wide racial variability in quality of care, particularly for Blacks and other underrepresented minority groups [3].

Similar disparities have also been observed in common post-stroke conditions–aphasia and dysphagia. Aphasia is a higher order disturbance of language that results in communication issues such as deficits in auditory comprehension verbal expression, reading, and writing that reduce an individual’s independence and quality of life [4]. Dysphagia is a swallowing disorder that can involve the oral cavity, pharynx, esophagus, or gastroesophageal junction, resulting in malnutrition and dehydration, aspiration pneumonia, and even death [5]. Approximately one-third of all stroke survivors experience aphasia [4] and approximately 50% experience dysphagia [6, 7]. Interestingly, stroke severity is not the sole factor that determines the presence or severity of post-stroke conditions such as aphasia and dysphagia as both conditions can occur after mild stroke. Both aphasia and dysphasia reflect injury to unique brain regions critical to language and/or swallowing and both conditions are independently associated with worse long-term outcomes, [7, 8] greater health services utilization, longer length of stays, and higher costs [9–12].

Regarding racial disparities in aphasia, our work has demonstrated worse outcomes among Blacks compared to Whites on measures of naming, word fluency, auditory word recognition, and comprehension of sequential commands based on a global aphasia assessment (Western Aphasia Battery-Revised) even after adjusting for age and years of education [13, 14]. Studies of dysphagia have also identified racial differences in outcomes. Bussell and colleagues found that Asian Americans, Blacks, and Native Americans experienced higher odds of having dysphagia than Whites [15]. Gonzalez-Fernandez et al. reported similar findings using state-level data [16]. Since stroke severity is not the sole factor of the presence or severity of aphasia and dysphagia, little is known about the cause of observed racial disparities in these two conditions.

Although it is generally recognized that health is a multidimensional construct and that outcomes are influenced by factors beyond an individual’s control (e.g., economic stability, health care access and quality of care, social and community context), prior research on aphasia and dysphasia has largely focused on symptom level comparisons of racial-ethnic groups. As such, there is a need for research accounting for the role of social determinants of health (SDoH) to better understand disparities in post stroke outcomes. SDoH are defined as “the complex circumstances in which individuals are born and live that impact their health. They include intangible factors such as political, socioeconomic, and cultural constructs, as well as place-based conditions including accessible healthcare and education systems, safe environmental conditions, well-designed neighborhoods, and availability of healthful food” [17]. Supporting the importance of the SDoH in understanding post stroke outcomes, Skolarus & Burke proposed a need to examine modifiable factors such as the medical, social, and community environment to better explain racial disparities [2].

To date, careful consideration of the complex range of factors that contribute to disparities in aphasia and dysphagia outcomes has yet to emerge. This is concerning given the complexity of rehabilitation care systems embedded within larger healthcare settings. Stroke rehabilitation care includes pre-hospital care, in-hospital care, and post-hospital rehabilitation—all of which contribute to post-stroke outcomes. Consequently, health disparities emerge from issues associated with each level of care (acute, subacute, outpatient) and during each interaction with healthcare providers. Thus, studies designed to examine disparities in clinical outcomes must consider the role of system level and individual provider level characteristics as well as the synergistic effect of subsystems and providers those patients utilize during the recovery process are needed. Therefore, this study will identify the factors that impact both the receipt and quality of rehabilitation care in acute, post-acute, and post-discharge (community) settings.

Previous studies, including our own work, suggest disparities in aphasia and dysphagia outcomes are not simply the result of the *quantity and timing* of services provided in the post-acute and community settings. We posit that these disparities can be better understood and potentially mitigated using a more comprehensive model. We aim to determine the association between outcome disparities between Blacks and Whites and the quality of rehabilitation care. By identifying and quantifying the receipt of quality rehabilitation care and identify the specific points along the rehabilitation continuum of acute care, post-acute care, and the post-discharge (community) environments, will can identify barriers to optimal care and how they contribute to disparities. Our central hypothesis is that system and provider factors (barriers) contribute to variations in the location, timing, and intensity of rehabilitation care received by stroke survivors with aphasia or dysphagia across the continuum of care. We propose to use decomposition methods to better understand the drivers of healthcare disparities. We propose that risk factors including healthcare institutional practices as well as community factors or social determinants of health such as residential housing, socioeconomic status, the criminal justice system, local school quality, and workplace environment indirectly influence health-related outcomes and the observed disparities. The specific aims of the project are as follows:

1. To determine how availability, accessibility, and quality of post-stroke acute inpatient care contribute to disparate outcomes of individuals with aphasia and dysphagia.
2. To determine how the timing and transition of care contribute to disparate outcomes of individuals with aphasia and dysphagia.
3. To determine how post-discharge community environments contribute to disparate outcomes of individuals with aphasia and dysphagia.

## Materials and methods

This study has been reviewed and the team received written approval from the University of Florida Institutional Review Board (IRB). Medicare data utilized in this study includes non-identifiable and identifiable files. Identifiable files are approved by the CMS Privacy Board and include only minimum data necessary to protect the beneficiary’s privacy. CMS also requires university IRB approval specifically for identifiable files. Files will be housed on ResVault which is UFs repository for restricted data being utilized for research purposes.

### Data

Our data will be drawn from a 100% sample of Medicare fee-for-service claims for persons hospitalized for a first-time stroke. The index hospitalization will be defined as an acute care hospital admission for a first-time stroke. Individuals with post-stroke aphasia or dysphagia will be identified based on their respective ICD-10 codes; aphasia (R47.01) and dysphagia (R13.1). The acute care hospital admission is considered the index hospital regardless of whether they were transferred from other facilities because of the importance of stroke-related discharge planning. Patients will be followed for at least 12 months after the index discharge to measure the timing of post-acute care transition(s) and post-acute care SLP utilization. Severity of stroke will be based on metrics developed by Simpson and colleagues [18] using documents comorbidities. To observe utilization across the continuum of care we will limit the sample to Black and White beneficiaries who were continuously enrolled in Medicare Part A and Part B under the Old Age and Survivors Insurance (OASI) or the Disability Insurance Benefit. The data include all fee-for-service facility and professional claims and aphasia/dysphasia assessments occurring in post-acute care facilities. Medicare data offers a unique opportunity to explore stroke care as 75% of stroke occur in individuals over the age of 65 [19] and 65% of individuals experiencing a stroke use Medicare as a primary payer source [20]. CMS claims, assessment, and enrollment files will be purchased from Research Data Assistance Center (ResDAC) [21]. Data on acute care hospitals, rehabilitation hospitals, skilled nursing facilities, and home health agency characteristics will be drawn from the CMS Cost Reports [22]. Acute Care Hospital Quality Data will be collected from annually published CMS public reports on stroke care performance as part of its Hospital Compare program [23]. We will obtain performance data on risk-adjusted 30-day stroke mortality; 30-day stroke readmission rates; and a measure emergency department (ED) performance on OP-23, which measures the percent of stroke patients who receive a head computed tomography (CT) or magnetic resonance imaging (MRI) scan interpretation within 45 minutes of arrival at the ED.

### Key variables

Measurement of aphasia/dysphagia service utilization, intensity of care, and outcomes will be determined using Current Procedural Terminology (CPT) and Healthcare Common Procedure Coding System (HCPCS) codes. CPT and HCPCS codes are five-digit numeric and alphanumeric codes assigned to services provided accompanying surgical or diagnostic procedures. These codes are used by Medicare to determine reimbursement amounts for services provided in outpatient settings and to document service provision in facilities.

### Outcomes

Aphasia/dysphasia treatment initiation will be measured as any claim for a person with an ICD-10 code for aphasia (R47.01) and dysphasia (R13.1) and a CPT code for SLP treatment within 60 days of the index discharge for those discharged home, or documentation of an SLP in the assessment data for those discharged elsewhere (IRF, SNF, Swing Bed, and HH). The length of time for those at home will be measured using the date of service in the claim. The number of sessions, measured as a count of the number of distinct sessions for evaluation and treatment of aphasia/dysphagia, will be identified using the CPT codes dedicated to speech-language pathology. Improvement will be measured as continuous and categorical (i.e., 0 = no change; 1,2,3 = small, moderate, large change, respectively) to account for differences in the scale of scores. These outcomes will be captured using the National Outcomes Measures (NOMS) [24, 25], the Outcome and Assessment Set (OASIS) for HH [26, 27], the Minimum dataset (MDS) for SNF, and the Inpatient Rehabilitation Facility—Patient Assessment Instrument (IRF-PAI) [28]. NOMS, OASIS, MDS, and IRF-PAI will also be utilized to measure change in the functional status (aphasia/dysphagia outcomes) at each level of post-acute care. Distance to treatment will be computed as the distance between the epicenter of the individual’s zip code and each acute care hospital and post-acute care type using the Open-Source Routing Machine algorithm available in Stata [29, 30].

Local Dissimilarity and Demographic Characteristics will be developed from Census tract-level data in the American Community Survey (ACS) on income, poverty rates, health insurance, education, institution, and housing characteristics by race and used to create dissimilarity indices. We will calculate indices of: school dissimilarity (neighborhood deprivation index); housing dissimilarity; educational dissimilarity (relative proportions of Blacks to Whites who were employed, employed in professional occupations, and attained a bachelor’s or higher degree); incarceration dissimilarity (prison incarceration and juvenile custody rates); poverty dissimilarity (Black to White ratios of median household income); primary care dissimilarity (disparities in health insurance); and employment dissimilarity (disparity in unemployment rates) [31]. Data will be aggregated to the zip code level using an existing crosswalk [32] and merged with patient data.

### Sample size considerations

National estimates have shown that between 2020 and 2022 the age-adjusted prevalence of stroke among those aged 65 and above was 7.7 (CI = 7.5–7.9) compared to 3.8 (CI = 3.6–3.9) and 0.9 (CI = 0.8–1.0) among those aged 45 to 64 and 18 to 44, respectively [33]. These estimates support the use of Medicare claims for this study given the higher prevalence of stroke among those over age 65. Furthermore, they provide a benchmark for validation of our findings, assessment of our cohort, and contextualization of the observed health services patterns.

We will study complete care episodes occurring between 2016–2019 providing a sample size of about 3.21 million. We expect to have a full year of follow-up data for about 2.41 million persons whose stroke hospitalizations occurred between 2016–2018 and will exclude episodes that were not completed within 2019. We will limit 2016 data to episodes that began in the second half of 2016, leaving us with about 2 million episodes for which we have at least 6 months pre- and 12 months post-stroke data. We expect an initial sample of stroke survivors that will consist of 370,000 who have aphasia and 660,000 will have dysphagia. Some individuals will have both conditions. The estimated sample size based on the above criteria is 802,441 patients, of which 96,821 (12%) will be Black. This sample will be large enough to detect statistically significant racial differences if they exist [34]. We acknowledge that biases that might exist regarding how data were obtained, recorded, and reported to Medicare or be created when patients change providers within systems or move from system to system [35]. Our team includes researchers with substantial experience with Medicare data who will consider all relevant confounders when conducting analyses and interpreting results and utilize strategies that minimize error.

Potential Data Concerns: While Medicare claims files provided by CMS for research purposes provide an innovative mechanism for evaluating the post-stroke continuum of care, they may introduce unavoidable biases and error related to receipt of care, diagnoses, medical coding, and clinical information.

#### Receipt of care

Conditions must be diagnosed to appear in the utilization files; however, some diseases and/or conditions are often under-diagnosed. In addition, while the files provide a reliable record of the care received by the beneficiary, they do not provide information on the care *needed*. This may make it difficult to study recurrence since the data only reveal the receipt of a service or treatment. Additionally, services that providers know in advance will be denied may be inconsistently submitted as bills and, therefore, inconsistently recorded in the files. Covered services for which claims are not submitted are not included in the data nor are services that may be received but are not covered by Medicare. Finally, research suggests that beneficiaries will either very high or very low number of annual encounters may be the result of record duplication, misfiling, or clerical mistake. Therefore, all outlier records will be assessed for validity and potential exclusion. *Diagnoses*: The data do contain information on chronic diseases; however, the data do not indicate the length or severity of their condition. Medicare claims provide limited precision in identifying diagnosis, especially for identifying condition subtypes. The sensitivity rate of claims may vary by year and location. Therefore, findings will not be reported at the granular level. It is not possible to verify diagnosis information presented within claims files making false positive and false negative identification difficult to identify. To account for these subsets of cases, estimates will always be presented along with error rates. Furthermore, the study team will not attempt to impute missing diagnostic information since the validity of these imputations cannot be tested.

#### Coding

Different care settings use different coding systems for procedures treated in inpatient and outpatient settings. Furthermore, hospital outpatient care is coded as a mix of CPT and revenue center (hospital billing center) codes. Currently, there exists a less-than-perfect crosswalk between codes. Since the specificity of coded data may vary temporally and spatially, these aspects of the data will be highlighted in reports of the study findings.

#### Clinical information and timing

Physiological measurements such as blood pressure, pulse, and cardiac ejection fraction are absent from the utilization files. Exact timing of events can be difficult to discern. Specifically, the time from admission to a given event or timestamps for dates of service cannot be found in the data. Additionally, encounter data do not include information on payments to providers.

### Statistical analysis approach

Measurement of Health Disparities using Regression Decompositions: This study uses regression decomposition methods, designed to “understand the multiple factors that contribute to underlying health disparities” [36]. Decomposition methods can be conceptualized as counterfactual comparisons that contrast an observed outcome with hypothetical outcomes which are estimated using different scenarios about the factors contributing to disparities. For example, this method uses a regression framework to determine if outcomes are different between racial groups with the same risk factors, holding other factors constant. Studies of conditions such as aphasia and dysphagia must consider that each condition requires complex management of care that frequently occurs in multiple settings. As such, measures that focus on one point in time (e.g., acute care) will not adequately account for the impact that each level of care has on the outcomes. Disparities in outcomes do not emerge from one static event; they are based on systemic and organized decisions which may not be obvious to healthcare systems and providers [37]. Therefore, this project has been designed to explore treatment received across multiple timepoints to determine how they contribute to short- and long-term aphasia/dysphagia outcomes.

Regression decomposition measures the extent to which differences between the outcomes of two groups are explained by 1) differential endowments (or characteristic), such as geographic access, education, risk factors, or insurance coverage; or 2) differences in decision-making, defined as between group differences in outcomes despite equal endowments or unmeasured differences [38–45]. For example, a simplified analysis of income differences between Groups A and B would posit that the differences can be explained by different education levels: *(1) IncomeA–IncomeB = αA−αB+ (βA EducationA−βB EducationB) + εA−εB*. Assume Group A consists of a higher proportion of highly educated individuals than Group B. If the estimates revealed that *α ^A = α ^B* and *β ^A = β ^B* then differences in income would be entirely explained by differences in education attainment (i.e. endowments) because (1) simplifies to (1’) *IncomeA–IncomeB = β ^A (EducationA− EducationB)*. However, if in addition to difference in education levels, the estimated coefficients also revealed that *β ^A > β ^B* then (1) becomes (1”) *IncomeA–IncomeB = (β ^A EducationA−β ^B EducationB)*. This result would imply that not only is Group A endowed with higher educational attainment, but they also benefit from preferential decision-making regarding the relationship between education and income. Stated more simply, the finding that a member of Group A with identical educational endowment is rewarded with higher income than a member of Group B is consistent with a disparity caused by structural barriers—Group A benefits from preferential decision-making about the value of their education. A finding that α ^A>α ^B may be evidence of structural barriers influencing outcomes but may also be due to unmeasured differences in endowments (i.e., confounders).

In Aim 1, we will test whether there are Black-White differences in the quality of admitting acute care hospital and inpatient spending on aphasia/dysphasia treatment using post-stroke inpatient hospitalizations. We will use regression decomposition to understand whether racial differences (or lack thereof) are due to variations in endowments or decision-making. In Aim 2, we will decompose Black and White differences in post-acute care timing and location to understand the role of structural barriers. Regression decomposition will identify the portion explained by variation in patient and disease characteristics, local post-acute characteristics, and/or acute care hospital types (i.e., endowments) versus differences in the relationship between the endowments and the outcome (i.e., decision-making). In Aim 3, we will examine the contribution of post-acute care and post-discharge community environments on aphasia/dysphasia outcome disparities and identify the factors that contribute to disparities. More specifically, local barriers to healthcare system access and local community dissimilarities will be assessed to measure their contributions to disparities in outcomes. The overall goal of this study is to understand whether racial disparities in where patients are treated, how they are treated, and where they reside can explain differences in aphasia and dysphasia outcomes.

The analyses will be conducted using Stata 18.0 or higher [46] and utilize a decomposition approach based on the original Blinder-Oaxaca Decomposition [47] which was based on ordinary least squares regression assuming linear associations and additive effects. More recently, methods have been developed to perform analogous decompositions of nonlinear specifications, including logit/probit, multinomial logit, survival, and count data/duration models. These decomposition approaches have been increasingly applied to understand disparities in self-reported health, vaccination rates, obesity, and diet quality [48–53], but have rarely been used to study disparities in stroke treatment or outcomes.

## Discussion

The findings of this study will characterize the impact of institutional and provider practices on the receipt of quality rehabilitative care. Furthermore, these results will determine if and how these practices translate into racial disparities in aphasia and dysphagia outcomes.

### Limitations

This study utilizes Medicare claims. Some advantages of using claims data for analyses such as this include large, diverse sample sizes, longitudinal follow-up, lack of selection bias, and potential for complex, multivariable modeling. However, disadvantages include (a) the inherent limitations of claims data, such as incomplete, inaccurate, or missing data, or the lack of specific billing codes for some conditions; and (b) the inability, in some circumstances, to adequately evaluate the appropriateness of care. While we have designed our inclusion/exclusion criteria and analytic models to address and contend with these issues, all of our reported data analyses and findings will include a clear description of the data source and potential for unintended bias. Due to the presence of potential biases and other unobserved factors, the results of this study will not be interpretable as causal. While unmeasurable differences in Black-White treatment preferences will be estimated using a race-specific constant, it is not possible to account for the differences that remain after adjusting for covariates due to the myriad of potential factors. Therefore, results should be interpreted in the context of this sample group and are valid only for persons enrolled in Medicare.
